# Supplementary material for: The Association of MEG3 lncRNA with Nuclear Speckles in Living Cells
Source: Cells. 2022 Jun 16;11(12):1942. doi: 10.3390/cells11121942 (PMC9221825; doi:10.3390/cells11121942)
Supplement: Supplementary file 1 [file cells-11-01942-s001.zip › cells-1676799-supplementary.pdf]

Supplemental figures and movies

The association of MEG3 lncRNA with nuclear speckles in living cells

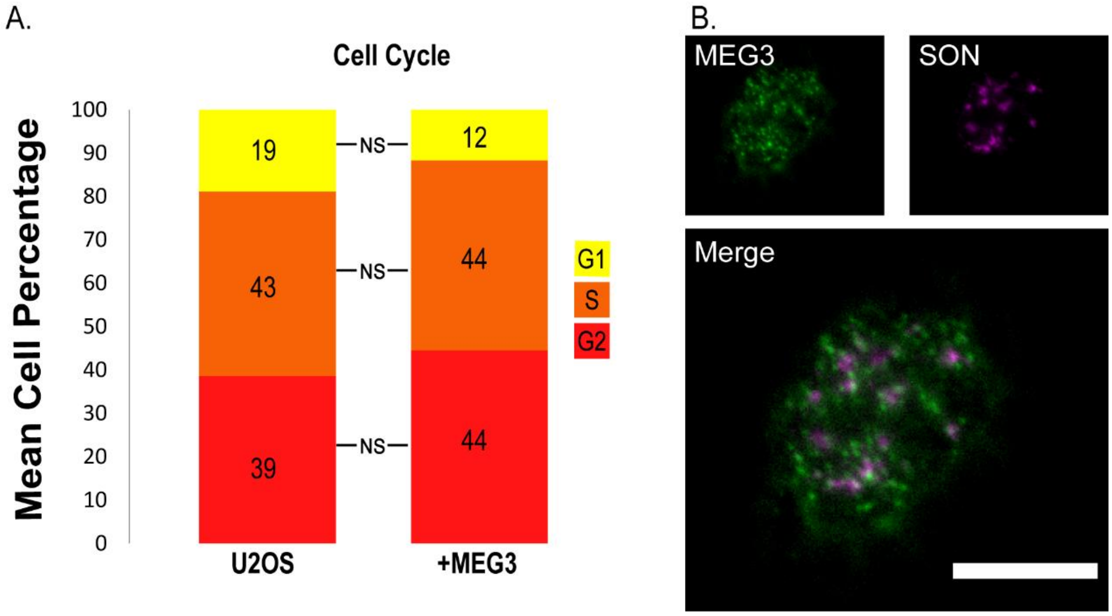

Supplementary Figure S1. Stable expression of MEG3 in U2OS Tet-On cells did not affect the cell cycle progression of the cells and had a similar expression pattern to endogenous MEG3. (**A**) FACS cell cycle analysis on wildtype U2OS cells (# cells = 21658, n = 3) and U2OS Tet-On cells expressing MEG3-MS2 transcripts (# cells = 25375, n = 3). Percentage of cells found in the different stages of the cell cycle: G1 (yellow), S (orange) and G2 (red) is depicted. The differences between the phases were analyzed using a *t*-test and found to be statistically insignificant (NS) ($p>0.1$). (**B**) HepG2 cells with RNA FISH of endogenous expression of MEG3 transcripts (green) and antibody staining for SON (magenta). Bar = 10 µm.

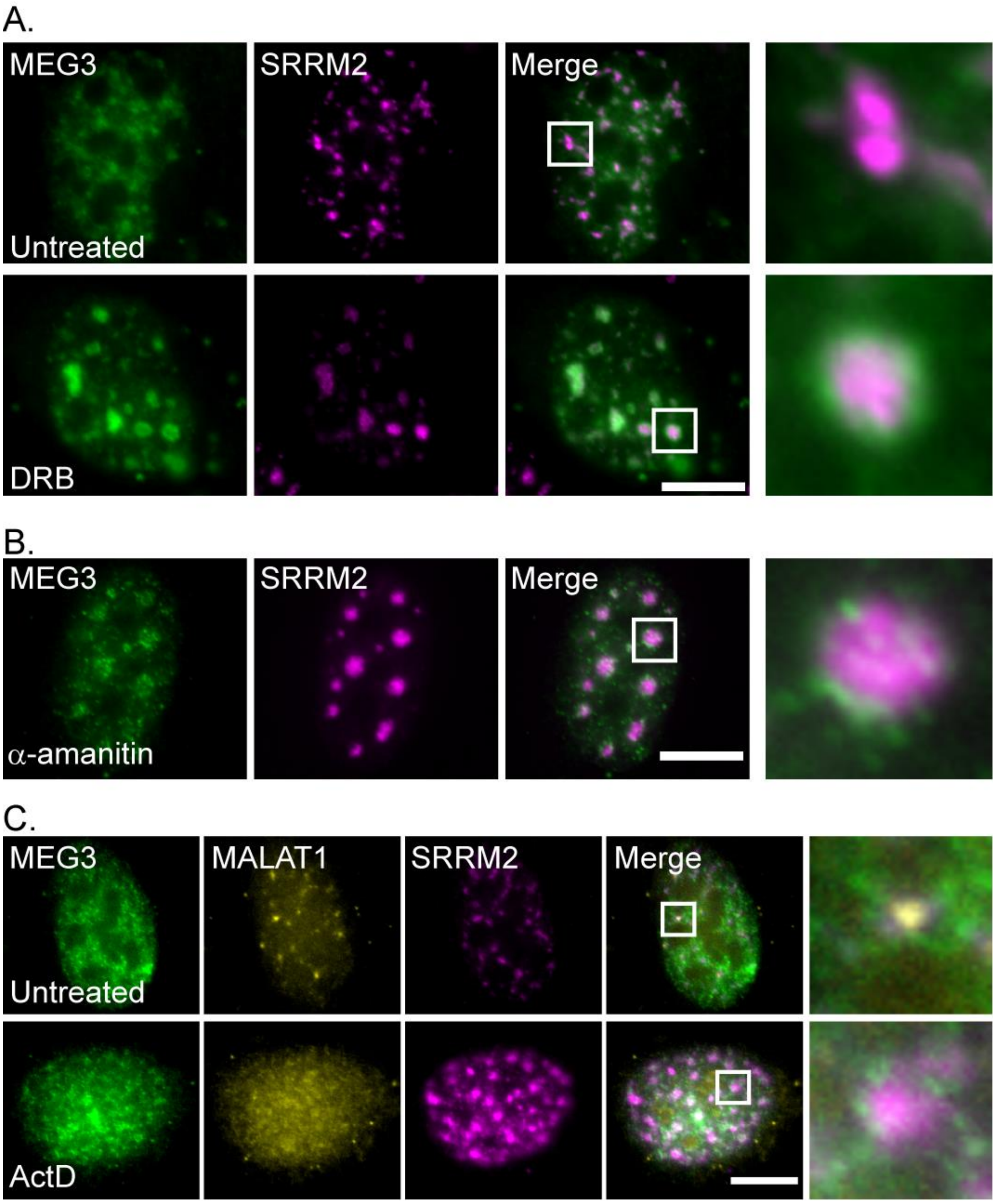

Supplementary Figure S2. MEG3 relocates to the nuclear speckles after transcription inhibition. (**A**) RNA FISH of MEG3 transcripts with fluorescent probes to the MEG3 sequence (green) in U2OS cells and antibody staining of SRRM2 (magenta) under transcription inhibition treatment with DRB for 2 hrs before fixation. Enlargements of the boxed regions appear on the right. (**B**) MEG3 YFP-MS2-CP tagged transcripts (green) and antibody staining of SRRM2 (magenta) transcription inhibition treatment with α-amanitin for 6 hrs before fixation.

Enlargements of the boxed regions appear on the right. (**C**) RNA FISH of MEG3-MS2 transcripts with fluorescent probes to the MS2 region (green) in U2OS cells together with endogenous RNA FISH of MALAT1 (yellow) and staining with an antibody to the nuclear speckle marker SON (magenta). (Top) untreated control cells and (bottom) cells treated with ActD for 2 hrs. Bars = 10 µm.

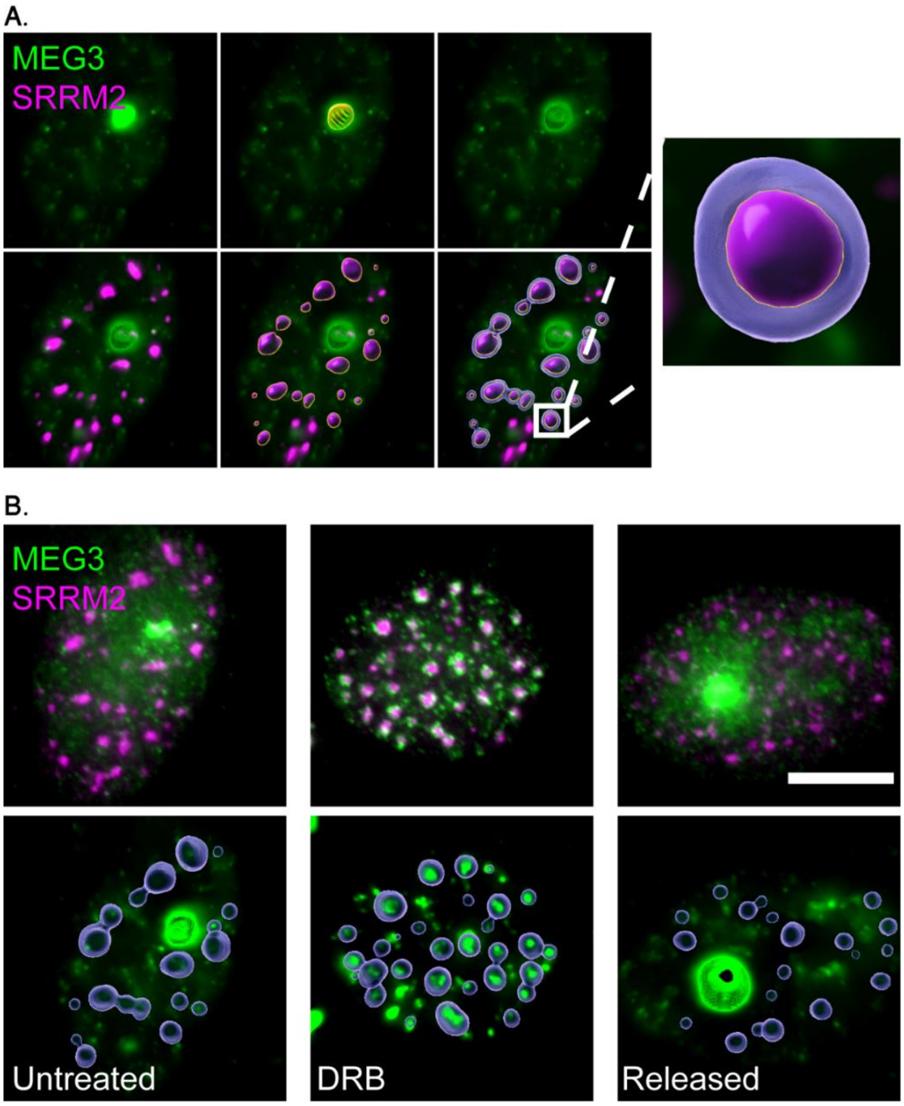

Supplementary Figure S3. Quantification of MEG3 association with nuclear speckles. Imaris software was used to analyze the MEG3 intensity at the nuclear speckle. (**A**) An untreated cell expressing MEG3 (green) where (top) the transcription site is detected, marked and removed from the analysis. (Bottom) The nuclear speckles (magenta) were then marked and a "bubble" (blue), extending with a threshold value of 0.25 in every direction from the surface of the nuclear speckle, was added. The intensity of MEG3 in the "bubble" was then measured. An enlargement of boxed area is on the right. (**B**) (Top) Original images and (bottom) final Imaris processed images of untreated (left), DRB treated (middle) and DRB-released (right) cells. Bar = 10 µm.

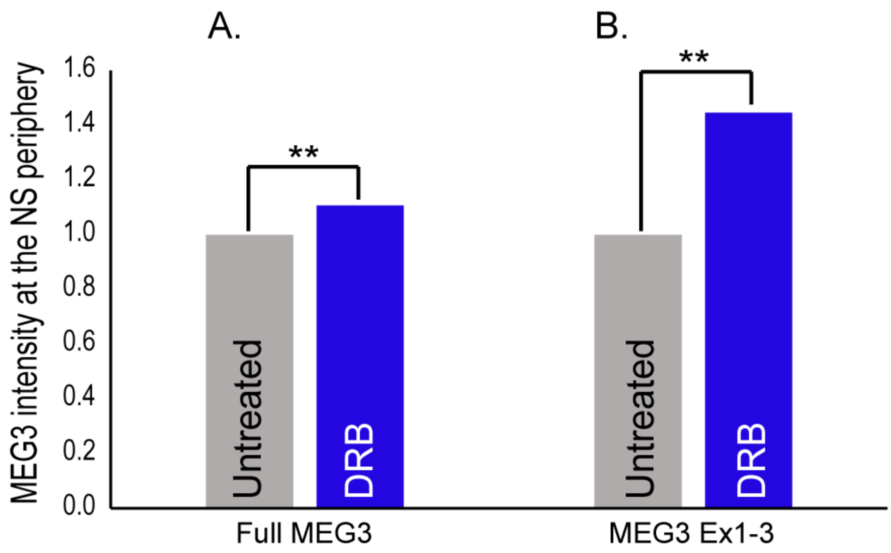

Supplementary Figure S4. Measurements of the changes in MEG3 intensity at the nuclear speckle periphery. (**A**) Measurements of the changes in MEG3 intensity at the nuclear speckle (NS) periphery under untreated conditions (#cells=94, #NS=1165) and DRB treatment (#cells=61, #NS=1031) in cells with the full MEG3 transcript. The graph shows the two first bars from figure 3C. (**B**) Measurements of the changes in MEG3 association with nuclear speckles under untreated conditions (#cells=10, #NS=108) and DRB treatment (#cells=12, #NS=97) in cells with the truncated transcripts MEG3 transcript containing exon 1-3 (**p<0.003). Statistical analyses of A and B were performed using a *t*-test.

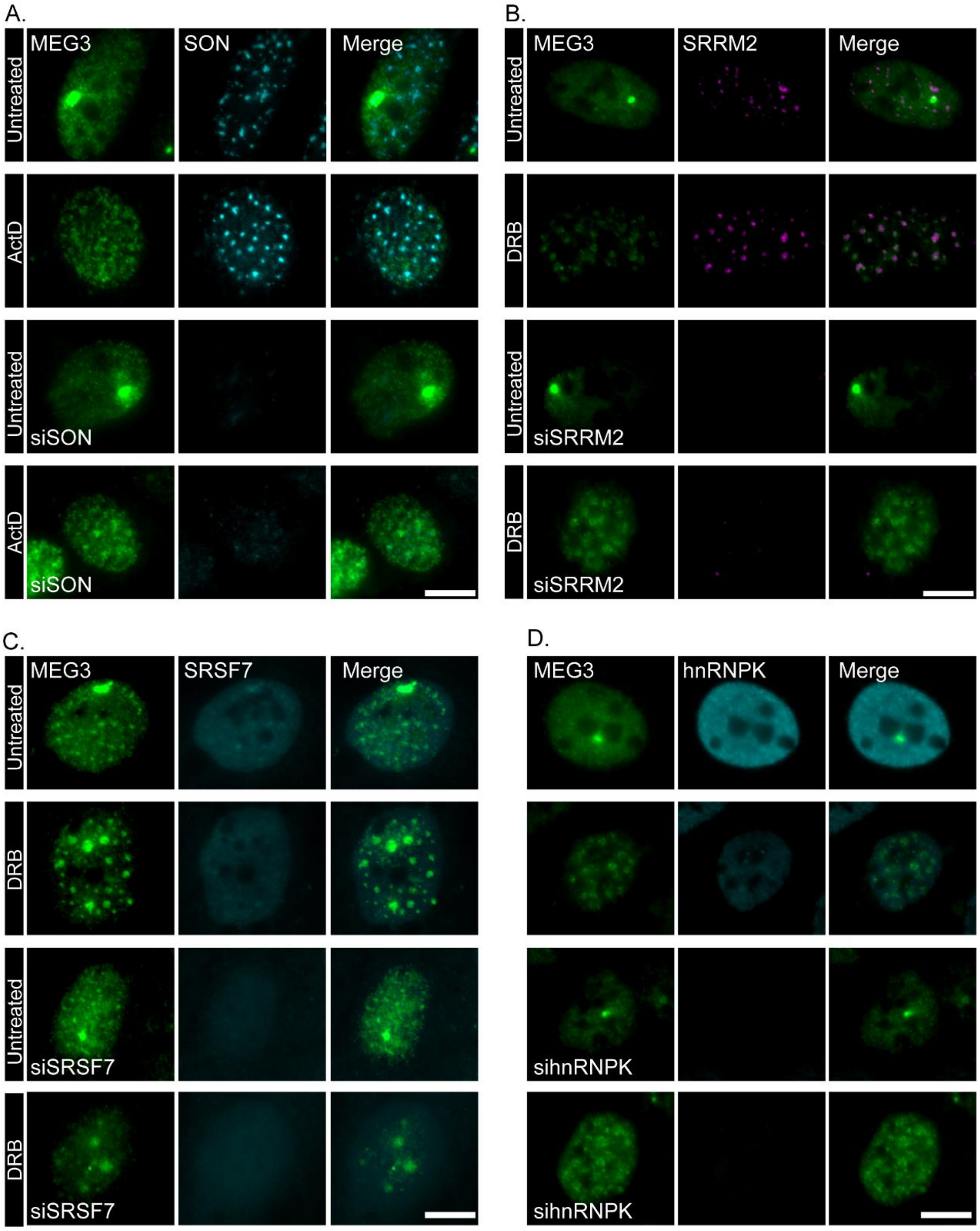

Supplementary Figure S5. Depletion of nuclear speckle proteins and RNA binding proteins does not change the association of MEG3 with nuclear speckles. Knockdown of (**A**) SON (cyan), (**B**) SRRM2 (magenta), (**C**) SRSF7 or (**D**) hnRNPK in cells labeled by RNA FISH for MEG3-MS2 transcripts (green). (Top) Untreated control cells, (top middle) cells treated with ActD or DRB

for 2 hrs, (bottom middle) siRNA knockdown, (bottom) siRNA knockdown with ActD or DRB treatment for 2 hrs. Bars = 10 μm.

Supplemental Movie Legends

Supplementary Movie S1. Live-cell imaging of U2OS cells stably expressing MEG3-MS2 tagged with YFP-MS2-CP after Dox activation (MEG3 grey signal is the inverted version of the original images). The small dots are MEG3 lncRNPs, and the large dot is the actively transcribing gene. There is a gradual increase of MEG3 signal over a 3 hr period. Imaged every 10 min for 3 hrs.

Supplementary Movie S2. Live-cell imaging of U2OS cells stably expressing MEG3-MS2 tagged with YFP-MS2-CP (MEG3 grey signal is the inverted version of the original images) where cells were treated with DRB 15 min before the start of the movie. Nuclear speckle association can be observed after about 40 min and remains throughout the movie. Imaged every 5 min for 1 hr and 40 min.

Supplementary Movie S3. Live-cell imaging of U2OS cells stably expressing MEG3-MS2 tagged with YFP-MS2-CP (MEG3 grey signal is the inverted version of the original images). The cells were treated with DRB for 2 hrs followed by release from the DRB immediately prior to the imaging. MEG3 is at the onset localized to the nuclear speckles and gradually disperses in the nucleoplasm. There is a complete loss of ring formation after 30 min of release from DRB. Imaged every 5 min for 1 hr and 20 min.

Supplementary Movie S4. FRAP live-cell imaging of an untreated U2OS cell stably expressing (left) MEG3-MS2 tagged with YFP-MS2-CP (yellow) and over expressing (right) Cerulean-SRSF3 (cyan). A red ring indicates the area bleached. MEG3 shows a clear nuclear speckle localization at the onset of the imaging and then there is recovery of the YFP signal at the specific nuclear speckle where the MEG3 signal was bleached.
